# Nutrient Regulation of Endocrine Factors Influencing Feeding and Growth in Fish

**DOI:** 10.3389/fendo.2019.00083

**Published:** 2019-02-28

**Authors:** Juan Ignacio Bertucci, Ayelén Melisa Blanco, Lakshminarasimhan Sundarrajan, Jithine Jayakumar Rajeswari, Cristina Velasco, Suraj Unniappan

**Affiliations:** ^1^Laboratory of Integrative Neuroendocrinology, Department of Veterinary Biomedical Sciences, University of Saskatchewan, Saskatoon, SK, Canada; ^2^Laboratorio de Fisioloxìa Animal, Departamento de Bioloxía Funcional e Ciencias da Saúde, Facultade de Bioloxía and Centro de Investigación Mariña, Universidade de Vigo, Vigo, Spain

**Keywords:** food intake, growth, nutrients, fish, aquaculture

## Abstract

Endocrine factors regulate food intake and growth, two interlinked physiological processes critical for the proper development of organisms. Somatic growth is mainly regulated by growth hormone (GH) and insulin-like growth factors I and II (IGF-I and IGF-II) that act on target tissues, including muscle, and bones. Peptidyl hormones produced from the brain and peripheral tissues regulate feeding to meet metabolic demands. The GH-IGF system and hormones regulating appetite are regulated by both internal (indicating the metabolic status of the organism) and external (environmental) signals. Among the external signals, the most notable are diet availability and diet composition. Macronutrients and micronutrients act on several hormone-producing tissues to regulate the synthesis and secretion of appetite-regulating hormones and hormones of the GH-IGF system, eventually modulating growth and food intake. A comprehensive understanding of how nutrients regulate hormones is essential to design diet formulations that better modulate endogenous factors for the benefit of aquaculture to increase yield. This review will discuss the current knowledge on nutritional regulation of hormones modulating growth and food intake in fish.

## Introduction

Physiological processes in fish, as well as in other vertebrates, are subject to complex regulatory mechanisms that act in response to both internal and external signals (1–4). Signals provided by the environment, along with internal cues are sensed and centrally integrated, providing information about the metabolic status. This enables fish to determine whether conditions are ripe to feed, grow, reproduce and save energy (4, 5). Among the external signals that can influence feeding behavior and growth in fish, one of the most important is food (6, 7). Both food availability and food composition exert a critical control of these processes, primarily by acting on the hormones in charge of their endocrine control. The main aim of this review is to summarize the recent advances on the role of feeding and fasting, as well as of dietary macro- and micronutrients, on the regulation of appetite- and growth-regulating hormones in fish. A better understanding of the effects of feeding status and diet composition on the expression and release of those hormones could be beneficial to determine the effects of a specific diet or feeding regime on fish health, growth, and development, which could be crucial in aquaculture. This review will also aim to identify gaps in knowledge and directions for future research regarding this important topic.

## Nutrients and Their Importance in Fish

Nutrients are organic compounds involved in biochemical reactions that produce energy and are constituents of cellular biomass (8). They are divided into two broad groups: macronutrients and micronutrients. Macronutrients are classified into carbohydrates (CHO), proteins and lipids, and are needed in relatively large amounts since they are the primary source to generate the energy organisms require to survive, grow, and reproduce. These nutrients can be stored within the body for later use, or be utilized, leading to the somatic growth of the animal (9). The micronutrients comprise of vitamins and minerals and are needed in smaller quantity, although they have several critical roles in cellular processes. The nutritional requirements regarding the composition and proportion of different nutrients present in the diet vary among species and within species, is determined by various extrinsic and intrinsic factors such as environmental conditions, stage of life cycle, sex, and reproductive state (10). The importance and main roles of each nutrient in fish metabolism are summarized in Table 1.

**Table 1:** The role of principal macronutrients in fish metabolism.

**Nutrient**	**Major role in fish**	**Importance for fish metabolism**	**References**
*Carbohydrates (CHO)*	Stored as glycogen that can be mobilized to satisfy energy demands when necessary. In fish, CHO seems to play only a minor role compared to lipids and proteins. Fish are in general unable to rapidly lower circulating glucose levels following a glucose load or a high CHO meal, thus leading to the interpretation that fish are glucose-intolerant.	Great importance to the metabolism of all fish species, since they act as an oxidative substrate to some cells and tissues. Control of glucose levels exists in tissues relying on glucose such as the brain. Numerous studies carried out in recent years have also demonstrated the existence of glucose sensing mechanisms in such tissues.	(7, 11–14)
*Lipids*	Storage and provision of metabolic energy in the form of ATP provided through the β-oxidation of fatty acids. They also play an important role as precursors for the synthesis of many hormones and in the formation of cell membranes.	Together with their constituent fatty acids, they are the main nutrients playing important functions as sources of metabolic energy for growth and reproduction. In fish diet, they are particularly important the *n*-3 and *n*-6 unsaturated fatty acids (“omega 3” and “omega 6”), which are not synthesized by animals, and therefore must be supplied in the diet.	(15–21)
*Proteins*	Amino acids are an essential component of the diet of all animals. Fish require a balanced combination of the 20 amino acids, of which 10 are not synthesized by fish and adequate amounts must be provided through their diet. These essential amino acids are methionine, arginine, threonine, tryptophan, histidine, isoleucine, lysine, leucine, valine and phenylalanine.	Proteins and amino acid requirements differ between fish species since they develop different roles. Some proteins are enzymes, catalyzing a wide range of chemical reactions; other proteins have essential functions in muscle contraction, the transport of specific molecules or as structural elements. In carnivorous fish, proteins also have an important role as a source of energy.	(9, 21, 22)
*Vitamins*	Are necessary for normal fish growth and health. They are usually not synthesized by fish and must be supplied in the diet. Vitamin deficiency results in scoliosis, dark coloration and/or, most commonly, reduction in growth rate.	Are highly important in the proper functioning of the metabolism since some of them are enzymatic co-factors.	(20)
*Minerals*	Fish can absorb many minerals directly from the water through their gills and skin, allowing them to compensate in some degree, mineral deficiencies in their diet. Some are incorporated into the bones, while others have a major function in acid-base balance, electron transfer or the maintenance of cell homeostasis.	They can be divided into two groups based on the quantity required in the diet. Macro-minerals such as sodium, chloride, potassium or phosphorous, regulate osmotic balance and are integrated into the skeletal structure. Micro-minerals are required in small amounts as components in enzyme and hormone systems.	(20, 21, 23)

Organisms coordinate their growth and development with nutrient level fluctuations in their environment, and therefore they must be able to sense their internal and external nutrient levels (24). In mammals, sensing mechanisms regulate specific processes such as food intake, hormone secretion, and energy expenditure to maintain energy homeostasis (25–27). The sensing of nutrients may occur directly or indirectly. In the first case, the nutrient molecule binds to its sensor. In the second case, nutrient abundance is detected through a related molecule (8). These detection processes occur in both central and peripheral tissues in fish. At the central level, the brain integrates metabolic information related to nutrient availability, satiety/hunger signals and hormones related to adiposity. As a result of such signal integration, a response is generated in peripheral tissues aiming to modulate the metabolism (28, 29). At the periphery, metabolic regulation by the sensing systems occurs directly or indirectly through the action of endocrine effectors (28).

## Hormones Regulating Food Intake

The regulation of food intake involves the integration of exogenous and endogenous factors to supply the energy necessary to support biological processes. Such regulation is achieved by the endocrine system, which secretes hormones and regulates the activity of the cells by transferring information between the organs. The major organs that secrete hormones involved in regulating appetite are the brain (hypothalamus) and gastrointestinal tissues. The following section will provide a brief description of the main hormones with a critical role in feeding regulation.

### Brain Hormones/Neuropeptides

#### Neuropeptide Y (NPY)

Neuropeptide Y, a 36 kDa amino acid protein, belongs to the NPY family of peptides, which also includes the pancreatic polypeptide (PNP) and peptide tyrosine (PYY). All these peptides share a common three-dimensional structure composed of polyproline coil and an amphipathic helix. The structure of the neuropeptide Y family is tightened by hydrophobic interactions between prolines and the helix (30). NPY is mainly secreted by the neurosecretory cells in the hypothalamus and is abundantly expressed in the brain, pituitary, spleen, gastrointestinal tract, kidney, testis and smooth muscles (31, 32). In teleosts, NPY has been shown to play an essential role in stimulating feeding [see Volkoff (33) for review].

#### Agouti Related Protein (AgRP)

AgRP is a 128 amino acid neuropeptide released by the NPY/AgRP neurons, and is an endogenous antagonist of melanocortin receptors 3 and 4 (MC3R and MC4R) (34). It is mainly expressed in the brain, but it is also found in several peripheral tissues, including ovary, muscle and ventral skin (34). AgRP receptor is highly localized at the site of the paraventricular nucleus, the dorsal motor nucleus of the vagus nerve and also in the raphe nucleus, all areas that are highly involved in energy homeostasis. AgRP acts as an orexigenic factor in fish, by antagonizing the activity of MC4R (33).

#### Proopiomelanocortin (POMC)

POMC is a 267 amino acid peptide secreted by the hypothalamic neurons located in the arcuate nucleus, as well as the corticotropic cells of the anterior pituitary, the melanotropic cells of the pars intermedia and skin melanocytes (35). In vertebrates, precursors of POMC has three domains, namely N-terminal pro-γ-melanocyte stimulating hormone (MSH), adrenocorticotropic hormone (ACTH) and C-terminal β-lipotropin, which are cleaved by the action of prohormone convertases. The most important of these derivatives are α-MSH, which plays a vital role in suppressing feeding by acting as an agonist at the anorectic MC4R (33), and ACTH, which regulates the secretion of glucocorticoids from the adrenal glands (36, 37).

#### Cocaine- and Amphetamine-Regulated Transcript (CART)

CART was isolated from rat striatum upon injection of cocaine and amphetamine, two psychomotor stimulants (38). In goldfish, two forms of CART precursor exist, namely, CARTI that encodes a 117 amino acid pro-CART, and CART-II which encodes a 120 amino acid pro-CART (39). Both CART precursors have been reported to be abundantly expressed in brain, pituitary and also in other peripheral tissues such as eye, interrenal tissues, and gonads in goldfish (39). CART exerts multiple physiological functions in fish, including the inhibition of appetite (39), regulation of the stress response (40) and energy balance (41).

#### Orexins

Orexins/hypocretins consist of two orexins, orexin-A, and orexin-B, both cleaved from the same precursor, prepro-orexin (42, 43). In fish, both the prepro-orexin RNA and the peptides A and B have been shown to be abundant in the hypothalamus (44), as well as in the gastrointestinal tract (33). Two heptahelical G-protein coupled receptors are known to mediate orexin functions. Orexins have been reported to have a significant role in increasing feeding behavior and locomotor activity (33), and they were also implicated in the regulation of sleep, energy homeostasis and circadian cycle (45).

#### Melanin-Concentrating Hormone (MCH)

Melanin-concentrating hormone (MCH), a 17 amino acid cyclic peptide, was initially isolated from the brain of chum salmon (46). Two genes, MCH1 and MCH2, have been identified in zebrafish and pufferfish (47). The MCH receptor was identified as the GPCR SLC-1, later termed as MCH-R1. MCH-R1 couples to different G-proteins and plays an essential role in activating different signaling pathways. The MCH-R1 is preferentially expressed in the brain, particularly in the hypothalamus, areas of the cortex, arcuate and ventromedial nuclei, and olfactory lobes. MCH mainly acts on the melanophores regulating the color change and also lightens the skin in fish. Besides this, MCH seems to have a role in the regulation of feeding, although such a function is still unclear in fish [see Volkoff (33) for review].

#### Nesfatin-1

Nesfatin-1 is an 82 amino acid peptide originally isolated from the rat hypothalamus and is encoded in the nucleobindin-2 (NUCB2) gene (48). It is proposed that NUCB2 is cleaved by the prohormone convertases into three different peptides, namely nesfatin-1 (82 amino acids), nesfatin-2 (85–163 amino acids) and nesfatin-3 (166–396 amino acids), respectively. Of all these peptides, nesfatin-1 has been shown to have biological activity. The 30-amino acid mid-segment of nesfatin-1 is considered as the bioactive core and has been shown to affect appetite, the hypothalamus-pituitary-ovarian axis, and to modulate intracellular Ca2^+^ signaling in mammals (49). Among non-mammals, nesfatin-1 has been studied in various fish species, including goldfish (50), Ya-fish (51), and rainbow trout (52). In goldfish, nesfatin-1-like immunoreactivity has been found in the hypothalamus, particularly in the nucleus lateralis tuberis (NLT) (50). Exogenous administration of nesfatin-1 has been shown to cause anorectic actions in goldfish (50).

### Hormones Primarily Arising From Peripheral Tissues

#### Ghrelin

Ghrelin has been identified in numerous fish species. It consists of 28 amino acids in mammals and has 12 to 25 amino acids in fish depending on the species. The gene encoding the protein was identified within the chromosome 3 and consists of four exons. Ghrelin exerts its physiological functions by binding to the growth hormone secretagogue receptor-1a (GHS-R1a/ghrelin receptor) (53). This peptide is mainly released in the stomach (or its equivalent in stomachless species), although other tissues have been shown to synthesize the hormone, particularly the hypothalamus (54). The central role described for ghrelin is its potent orexigenic role (33), but there are other well-known physiological roles for this hormone, including the regulation of GH release from the pituitary, a role in energy balance regulation, and cardiovascular effects, among others (55–57).

#### Cholecystokinin (CCK)

CCK is a peptide characterized by a C-terminal tetrapeptide sequence. The structure of pro-CCK consists of three sulfated tyrosine residues, which play a crucial role in the activation of CCK receptors (58). CCK binds to two receptor subtypes, CCK-A receptor (CCK1) and CCK-B receptor (CCK2), which are primarily localized at the site of the gastrointestinal tract and the brain (59). In fish, CCK and its cleavage sites suggest that the precursor protein (pro-CCK) is processed into octapeptides and are fully sulfated. CCK plays an essential role in the regulation of feeding, influences digestion and activates satiety signals (60). In goldfish, acute administration of CCK resulted in the suppression of food intake (61), likely by acting on NPY and orexin-A (62).

#### Peptide YY

Peptide YY, a member of the Y family of peptides, is a 36 amino acid gut-brain hormone that is known to have anorectic actions in goldfish (63). PYY is released from the endocrine cells of ileum and colon (64), and binds to the NPY receptor 1 which is abundantly expressed in the brain and gut of fish. Similar to NPY, PYY plays an essential role in signaling between the enteric nervous system and central nervous system in fish (31, 65).

#### Glucagon-Like-Peptide-1 (GLP-1)

GLP-1, another anorexigenic intestinal peptide, belongs to the family of glucagon-like peptides encoded in the preproglucagon gene. In fish, the pancreas synthesizes glucagon and GLP-1, while the intestine secretes oxyntomodulin (66), and all of these peptides are processed from the proglucagon in the nervous system and intestine. GLP mRNAs have been identified in several teleosts, and its receptor (GLP-1R) has been successfully cloned in zebrafish and goldfish (67). Apart from reducing food intake, GLP-1 has been involved in gastric emptying and plays an important role in regulating liver glycogenolysis and gluconeogenesis (66).

#### Leptin

Leptin is a 16 kDa protein encoded in chromosome 7. While mammalian leptin is mainly produced by the adipose tissue, the liver appears to be the main leptin production site in fish (68). The structure of leptin resembles that of growth hormone, belonging to the family of tetrahelical cytokines. In fish, this hormone has been shown to affect adipogenesis (57), and to increase lipolysis while reducing lipogenesis in liver (69). Besides this, leptin decreases food intake in several fish species (33), likely by stimulating the anorexigenic neuropeptides POMC and CART (70).

## Hormonal Regulators of Growth

Regulation of growth in fish, as well as in most of the vertebrates, is coordinated by the GH-IGF system (71). A summary of each component of the GH-IGF system as well as their principal function related to growth is presented in this section.

### Growth Hormone

Growth hormone (GH) is an endocrine regulator of many physiological processes in vertebrates. In fish, GH is involved in almost all physiological processes including osmotic balance, lipid, protein and CHO metabolism, reproduction and growth. Moreover, studies have indicated that GH also affects behavioral aspects, such as appetite (72) and foraging (73) in rainbow trout and transgenic Atlantic salmon, respectively. In fish, GH is released from the adenohypophysis in response to hypothalamic signals, and exerts its effects on target tissues (74).

### Growth Hormone Receptors

In fish cells, GH binds to its receptors GHR-I and GHR-II (growth hormone receptor I and II, respectively) to exert its biological actions (75). GH receptors (GHRs) are widely distributed in tissues, but the primary expression is in the liver (or hepatopancreas). In that tissue, the important response to GH binding its receptors is the release of the insulin-like growth factor I (IGF-I). In other tissues, GHRs also mediate the growth-promoting effects of GH, although the liver is still the place in which GHRs have a significant role in the somatic growth regulation.

### Insulin-Like Growth Factors

As GHRs, IGF-I and IGF-II are expressed in several tissues, but the main expression is in fish liver. Both factors play a key role in the promotion of cellular proliferation and differentiation in vertebrates (76–78). These and other biological functions of IGFs are mediated by binding to specific transmembrane receptors, present in fish as well as in mammals (79). Apart from growth, IGF-I has also been associated with fish metabolism, development, reproduction and osmoregulation in seawater (74). The IGF-II mRNA has been detected in the liver as well as in the brain, heart, kidney, gills, gastrointestinal tract, pancreatic islets, skeletal muscle and gonads of fish (74). The widespread gene expression of *IGF-II* detected by RT-qPCR in both juvenile and adult fish contrasts the findings in mammals, in which its expression seems to be relevant only during the early stages of development (80). A role in metabolism regulation by IGF-II was demonstrated in muscle cells from trout (81), indicating that this factor could act as a metabolic hormone in fish. There is evidence that GH regulates *igf-II* gene in all tissues in fish (82–84), while GH most likely regulates only the expression of the *igf-I* gene in vertebrates. This situation makes fish an excellent model to study species-specific differences in the growth system.

## Nutritional Regulation of Hormones Modulating Food Intake in Fish

### Feeding

The nutritional status is an important modulator of appetite-regulating hormones in fish. In this respect, several central and peripheral appetite regulators are affected by a single meal, showing periprandial fluctuations in their expression and/or secretion levels. Brain hormones showing such changes in fish include NPY (85, 86), orexin (87–90), CART (87, 88, 91, 92), and nesfatin-1 (93). In general terms, appetite-stimulating or orexigenic factors, including NPY (85, 86), and orexin (89, 90), have been shown to display periprandial changes characterized by higher expression levels before or at mealtime, and lower levels post feeding, suggesting that they are hunger signals. By contrast, appetite inhibiting or anorexigenic signals, such as CART (91, 92), were found to be mainly upregulated after a meal, thus acting as a postprandial satiety signal.

As for peripheral appetite-regulating hormones, the most studied in terms of periprandial fluctuations have been ghrelin, CCK, PYY, and leptin. Circulating levels of ghrelin (in its acylated form) were found to rise pre-prandially in goldfish (94), supporting the role of acyl-ghrelin as a meal initiator in this teleost. Consistently, Unniappan et al. (95) described a significant decrease in total ghrelin in circulation after a meal in goldfish, although this postprandial decrease in plasma ghrelin levels seems to be species-specific as it was detected neither in rainbow trout (96) nor in Mozambique tilapia (97). Ghrelin has also been shown to display periprandial fluctuations in terms of gene expression, although different profiles have been described depending on the species [Atlantic cod, (90); gibel carp, (98); goldfish, (94, 95); Mozambique tilapia, (97, 99); *Schizothorax prenanti*, (100); zebrafish, (101)]. The periprandial changes of CCK have been only studied in terms of its mRNA expression. Although species-specific changes were observed (88, 89), periprandial variations of *cck* expression are mainly characterized by an increase in mRNA levels after a meal in the intestine and/or the hypothalamus [dourado, (87); *Schizottorax prenanti*, (102)]. A similar periprandial profile was described for *pyy* in the hypothalamus of the Mexican blind cavefish (89) and *Schizottorax prenanti* (103), in the brain of goldfish (63), and in the brain and gut of Siberian sturgeon (104). Finally, leptin seems only to vary postprandially, although such variations are species- and tissue-specific. Thus, postprandial increases in *leptin* mRNA expression has been observed in the brain and liver of the mandarin fish (105), in the liver of goldfish (106) and in the visceral adipose tissue of the Atlantic salmon (107), but not in the brain of goldfish (106), the brain and intestine of pacu (88), or the liver of *Schizothorax prenanti* (102).

### Food Deprivation

Food deprivation has been shown to regulate the secretion and expression of appetite-regulating hormones. Table 2 includes a summary of the available studies within the literature and shows the main effect of fasting periods of different duration on the circulating levels and the mRNA expression of the main appetite regulators in several fish species. In general terms, as expected, fasting has been found to upregulate the levels of orexigens and decrease the levels of anorexigens, but several exceptions have been observed depending on the duration of the fasting period, the tissue analyzed and the species (see Table 2 for results and references). In general an upregulation of orexigens and the GH-IGF system should result in an increase in growth. However, without a complete profile of the redundant endocrine milieu, such conclusions are not valid. In addition, a major limitation of many of the studies is that only mRNA expression was determined. Without understanding more about the peptide synthesis in its major tissue sources, and its levels in circulation, it is difficult to reach conclusions on the effects of nutrient status on these hormones.

**Table 2:** Effects of fasting periods of different duration on the circulating levels and the mRNA expression of the main appetite-regulating hormones in several fish species.

	**Short-term fasting** **(1–6 days)**			**Mid-term fasting** **(7–29 days)**			**Long-term fasting** **(≥30 days)**		
	**Circulating levels**	**Brain expres**.	**Periph. expres**.	**Circulating levels**	**Brain expres**.	**Periph. expres**.	**Circulating levels**	**Brain expres**.	**Periph. expres**.
NPY		Atlantic salmon = (108) Blunt snout bream ↑ (109) Goldfish ↑ (110)	Blunt snout bream = (109)		Blunt snout bream ↑ (109) Brazilian flounder ↑ (85) Coho salmon ↑ (111) Cunner ↓ (112) Nile tilapia ↑ (113) Platyfish = (114) *Schizothorax prenanti* ↑ (115) Tiger puffer ↑ (116) Winter flounder ↑ (117) Winter skate ↑ (118) Yellowtail ↑ (119) Zebrafish↑ (120)	Blunt snout bream = (109)		Rainbow trout = (121)	
AgRP		Atlantic salmon ↓ (108) Goldfish ↑ (34) *Schizothorax prenanti* ↓ (122) Sea bass ↓ (123)			Common carp ↓ (124) Goldfish ↑ (34) *Schizothorax prenanti* = (122) Sea bass ↑ (123) Zebrafish ↑ (125)			Rainbow trout = (121)	
POMC		Flatfish ↑ (126)			Goldfish = (127) Rainbow trout = ↑ (128) Zebrafish = (125) Zebrafish ↓ (129)			Rainbow trout ↑ (121)	
CART		Atlantic salmon ↓ (108) Catfish ↓ (130) Dourado = (87) Goldfish ↓ (39) Siberian sturgeon ↑ (92) Zebrafish ↓ (131)			Atlantic cod ↓ (132) Channel catfish ↓ (133) Common carp ↓ (124) Cunner ↓ (112) Goldfish ↓ (134) Pacu ↓ (88) Platyfish ↓ (114) Red-bellied piranha ↓ (135) *Schizothorax prenanti* ↓ (91) Siberian sturgeon↑ (92) Winter flounder ↓ (117) Winter skate = (118)			Rainbow trout = (121)	
Orexin		Dourado ↑ (87) Zebrafish = (136)			Blind cave fish ↑ (89) Cunner ↓ (112) Goldfish ↑ (134) Pacu ↑ (88) Platyfish ↑ (114) Red-bellied piranha ↑ (135) Zebrafish ↑ (136, 137)				
MCH		Starry flounder ↑ (138)			Atlantic code ↑ (139) Barfin flounder ↑ (140) Hammerhead shark = (141) *Schizothorax prenanti* ↑ (142) Winter flounder ↑ (143) Zebrafish ↑ (144)				
Nesf-1		Zebrafish = (93)	Goldfish ↑ (50) Ya-fish ↑ (51) Zebrafish = (93)		Goldfish ↓ (50) Ya-fish ↓ (51) Zebrafish ↓ (93)	Goldfish ↑ (50) Ya-fish ↑ (51) Zebrafish ↓ (93)			
Ghrelin	Atlantic salmon ↑ (145) Tilapia = (97)	Blunt snout bream ↑ (109) Grass carp ↑ (146) *Schizothorax prenanti* = (100) Zebrafish = (147) Zebrafish ↑ (148)	Atlantic salmon ↑ (149) Atlantic salmon ↓ (145) Blunt snout bream ↑ (109) Chinese perch ↓ (150) Gibel carp = (98) Grass carp ↑ (146) *Schizothorax davidi* ↑ (151) *Schizothorax prenanti* = (100) Sea bass = (152) Tilapia = (97) Zebrafish ↓ (153) Zebrafish ↑ (148)	Atlantic salmon = (145) Goldfish = (93) Hybrid striped bass ↑ (154) Rainbow trout = (96) Tilapia = (155) Tilapia ↑ (97)	Blunt snout bream ↑ (109) Goldfish = ↑ (93, 156) Grass carp ↑ (146) Red-bellied piranha = (157) Zebrafish = (147) Zebrafish ↑ (148)	Atlantic salmon = (145) Blunt snout bream ↑ (109) Gibel carp ↑ (98) Goldfish ↑ (93, 156) Grass carp ↑ (146) Red-bellied piranha ↑ (157) *Schizothorax davidi* ↑ (151) Sea bass ↑ (152) Tilapia = (97) Zebrafish ↑ (148)			
CCK		Blunt snout bream ↓ (109) Dourado = (87) Grass carp ↓ (158) *Schizothorax prenanti* ↓ (102)	Atlantic salmon = (149) Blunt snout bream ↓ (109) Dourado = (87) Grass carp ↓ (158) *Schizothorax prenanti* ↓ (102) White sea bream ↑ (CCK-1), ↓ (CCK- 2) (159) Yellowtail ↓ (160) Zebrafish ↓ (153)		Blind cave fish = (89) Blunt snout bream ↓ (109) Grass carp ↓ (158) Pacu = (88) Red-bellied piranha = (135) *Schizothorax prenanti* ↓ (102) Winter skate = (118) Platyfish ↓ (114) Cunner ↓ (112)	Blunt snout bream ↓ (109) Grass carp ↓ (158) Pacu ↓ (88) Platyfish ↓ (114) Red-bellied piranha = (135) *Schizothorax prenanti* ↓ (102) Winter flounder ↓ (117) Winter skate ↑ (118)	Cunner ↓ (112)		
PYY		Goldfish ↓ (63) Siberian sturgeon ↓ (104) *Schizothorax prenanti* ↓ (103)	Siberian sturgeon ↓ (104) Atlantic salmon = (149) Yellowtail ↑ (160)		Blind cave fish = (89) Goldfish ↓ (63) Nile tilapia ↓ (113) Red-bellied piranha = (135) Siberian sturgeon ↓ (104) *Schizothorax prenanti* ↓ (103)	Nile tilapia ↓ (113) Red-bellied piranha ↓ (135) Siberian sturgeon ↓ (104)			
Leptin		Mandarin fish ↑ (105)	Common carp = (161) Mandarin fish ↑ (105) *Schizothorax prenanti* ↓ (102)	Fine flounder ↑ (162, 163) Rainbow trout ↑ (164) Tilapia ↑ (1)	Goldfish = (106) Orange-spotted grouper = (165) Pacu = (88) Red-bellied piranha = (157) Tilapia = (166)	Common carp = (161) European sea bass ↑ (167) Goldfish = (106) Green sunfish ↓ (168) Orange-spotted grouper ↑ (165) Pacu = (88) Rainbow trout ↑ (169) Red-bellied piranha ↓ (157) *Schizothorax prenanti* ↓ (102) Striped bass ↓ (170) Tilapia ↑ (1) White-clouds Mountain minnow ↑ (171) Zebrafish ↓ (172)	Fine flounder = (163) Rainbow trout = (121)		Arctic charr ↑ (173) Atlantic salmon = (107) Eel = (174) Rainbow trout ↑ (121)

* = levels not altered; ↑ levels upregulated; ↓ levels downregulated*.

### Diet Composition

Another nutritional aspect influencing the appetite-regulating hormones is the composition of diets. This is of great importance as there is significant interest in fisheries and aquaculture in modulating fish growth and reproduction by altering diet and/or endocrine milieu. Therefore, it is important to understand the dietary regulation of hormones, as they have remarkable effects on both reproduction and growth. Although the literature available on this is not very large, several studies have described that altering the macronutrient (i.e., carbohydrates, proteins, and fat) composition of the diet has significant effects on the secretion and/or expression of appetite-regulating hormones in fish.

#### Carbohydrates (CHO)

Few studies have been performed in fish describing the effects of CHO on appetite regulators. In 2002, Narnaware and Peter described that feeding goldfish CHO-enriched diets significantly reduces NPY mRNA levels in brain areas (175). Additionally, this macronutrient has been shown to upregulate *preproghrelin* mRNA expression in the pituitary (176). An *in vitro* assay performed to test the effects of glucose on the expression of appetite-regulating hormones revealed that exposure of goldfish intestinal fragments to different concentrations of this monosaccharide leads to a downregulation of *nucb2/nesfatin-1* mRNAs and an upregulation of *preproghrelin* mRNA expression (177).

#### Proteins

High-protein diets also modulate important appetite-regulating hormonal systems. A study performed in sea bream revealed that fish fed on high protein diets has higher mRNA expression levels of *preproghrelin* and *cck* than those fish fed on diets containing a lower amount of protein (178). In goldfish, feeding diets enriched in this type of macronutrient results in a significant increase in *nucb2/nesfatin-1* expression in the pituitary, and a significant decrease in *nucb2/nesfatin-1* mRNAs in the gut and in *preproghrelin* mRNAs in the liver (176). Accordingly, expression of both *nucb2/nesfatin-1* and *preproghrelin* was reduced by direct exposure of goldfish intestinal and hepatopancreas fragments to L-tryptophan (177). Very recently, Volkoff et al. demonstrated that the replacement of dietary fish protein with soy protein does not produce major changes in the expression of *cart, orexin, cck*, and *leptin* in the pacu (88). Similarly, varying the diet lysine to arginine ratio has been described to not significantly modify the expression of *npy* and *cck* in juvenile cobia (86). Leucine reduced leptin secretion from the adipocytes of food-restricted rainbow trout (57).

#### Lipids

Intake of fat-enriched diets has been described to reduce the gene expression of *npy* in the goldfish telencephalon-preoptic area (175), and to increase the mRNA levels of *nucb2/nesfatin-1* in the hypothalamus and liver, and of *preproghrelin* in the pituitary of goldfish (176). Treatment of goldfish intestine with different fatty acids *in vitro* revealed that fatty acids, in general, downregulate NUCB2/nesfatin-1 in the intestine, but only the longer and highly unsaturated fatty acids inhibit preproghrelin (177). Jönsson and coworkers showed that rainbow trout fed a normal-protein/high-lipid diet tends to have higher plasma ghrelin levels than those fed a high-protein/low-lipid diet (96). Apart from the above-mentioned studies, which were all carried out in adult fish, few studies have been performed at larvae or post-larvae state to study the effects of the replacement of the dietary fat source on the expression of metabolic hormones. Bertucci and coworkers demonstrated that the replacement of dietary fish oil with sunflower oil leads to a decrease in *nucb2/nesfatin-1* mRNA expression in pejerrey larvae (179). Additionally, it was described that Senegalese sole post-larvae fed with diets containing soybean oil have higher *cart1* and *cck* mRNA levels in the brain, but lower peripheral *cck* levels than larvae fed cod liver oil (180).

## Nutritional Regulation of the GH-IGF System and Its Influence on Fish Growth

### Nutritional Status

The main environmental factor that regulates the GH-IGF system is the nutritional status (181). Table 3 summarizes the effects of fasting on the expression of components of the GH-IGF system in different fish species. During fasting, growth ceases, and energy is mobilized from tissues to support metabolism. This is mainly mediated by changes in the GH-IGF system: plasma levels of GH generally increase while plasma levels of IGFs decrease (182). These changes are explained by a phenomenon known as liver GH resistance, which is characterized by the fact that hepatocytes become resistant to GH, resulting in decreased IGF production despite elevated GH (197). These changes in GH-IGF system during fasting could be adaptive jn response to the high GH plasmatic levels together with low levels of IGF-I (and also insulin) induces lipolysis making fatty acids available to peripheral tissues (197). Using this mechanism, fish are capable to usually maintain its internal functions during fasting, avoiding somatic growth.

**Table 3:** Effects of fasting periods of different duration on the circulating levels and the mRNA expression of the GH-IGF system endocrine components in several fish species.

	**Short-term fasting** **(1–6 days)**			**Mid-term fasting** **(7–29 days)**			**Long-term fasting** **(≥30 days)**		
	**Circulating levels**	**Brain/Pit expres**.	**Periph. expres**.	**Circulating levels**	**Brain/Pit expres**.	**Periph. expres**.	**Circulating levels**	**Brain expres**.	**Periph. expres**.
GH	Chinook salmon = (182) Tilapia ↑ (97)			Channel catfish = ↑ (183) Chinook salmon ↑ (182) Coho salmon ↑ (76, 184) Fine flounder ↑ (162) Rainbow trout ↑ (185–187) Tilapia ↑ = (97, 188, 189)	Channel catfish = ↑ (183) *Cichlasoma dimerus* = (190) Crucian carp ↓ (191) Grouper ↑ (192) Tilapia ↑ (188)				
IGF-I	Chinook salmon = ↓ (182) Tilapia ↓ (97)		Chinook salmon = ↓ (182)	Channel catfish ↓ (183) Chinook salmon ↓ (182) Coho salmon ↓ (184) Rainbow trout ↓ (185) Tilapia ↓ (97, 188, 189, 193)		Atlantic salmon = (145) Channel catfish ↓ (183) Chinook salmon ↓ (182) Coho salmon *Cichlasoma dimerus* = ↓ (190) ↓ = (76) Crucian carp ↓ (191) Grouper ↓ (192) Tilapia ↓ (188, 189, 193) Yellowtail = (194)	Brown trout ↓ (195) Masu salmon ↓ (196)		Masu salmon = (196)
IGF-II						Atlantic salmon = ↓ (145) Tilapia = (193)			

= levels not altered; ↑ levels upregulated; ↓ levels downregulated

Studies on the effects of feed quantity are available regarding the GH-IGF system. In general, increased feed ration results in inverse changes in GH-IGF system hormones, compared to fasting (182, 198–200). This state in which plasmatic GH levels are low and IGFs are high is correlated with an increase in the somatic growth rate of fish (200). However, if feed ration size is considerable, plasmatic GH levels are high, and GHRs and IGFs levels remain low. This probably diminishes feed utilization for growth (199).

### Diet Composition

#### CHO

Diet composition is another important factor regulating the GH-IGF system and somatic growth (197). Several works have been carried out in the past years aiming to determine the effect of dietary carbohydrates, especially glucose, on GH in fish. Rodgers et al. (201) demonstrated that when tilapia pituitaries were incubated in the presence of varying glucose concentrations, the quantity of GH released is inversely related to glucose concentration in the culture media. However, Riley et al. (202) found no changes in pituitary GH mRNA or plasma GH in response to intraperitoneal (IP) glucose injection. In the liver, IP glucose treatment significantly elevates the levels of GHSR mRNAs. Although the IGF-I mRNA expression was not altered by the IP glucose injection, the IGF-I plasma levels were significantly reduced in tilapia (202). In an *in vitro* assay carried out by our group (203), it was found that incubation of goldfish hepatopancreas with different glucose concentrations significantly increases the expression of *ghr-I, ghr-II, igf-I*, and *igf-II* mRNAs at 4 h. The increase in GHR and GH mRNAs caused by glucose could be related its insulinotropic effect, as such outcomes were demonstrated in mammals (204). IGFs were postulated as regulators of glucose uptake in fish (205), and in mammals (206), likely through the modulation of GLUT-1 glucose transporter (207). GH has been shown to have a hyperglycemic effect in several species of fish, and it is glycogenolytic, glycolytic and gluconeogenetic in some tissues, including the liver, brain, and gill (208). Therefore, IGFs seem to play a role in maintaining the balance between GH and insulin to promote normal carbohydrate metabolism.

#### Proteins

Dietary protein seems to regulate the hepatic IGF-I expression and secretion, as it was demonstrated in mirror carp (209) and Nile tilapia juveniles (210). In both cases, authors found a significant correlation between dietary protein levels and hepatic IGF-I mRNA expression. Moreover, Qiang et al. (210) found that the increase in the dietary protein content not only increases the IGF-I mRNA expression but also increases the plasmatic IGF-I levels. These authors also reported a significant correlation between the fish-specific growth rate (SGR) and the IGF-I plasmatic levels and/or IGF-I hepatic mRNA expression. In gibel carp (211), it was found that an increase in dietary protein levels leads to an increase in SGR and hepatic IGF-I mRNA levels, although a decrease in both parameters was observed with extremely high protein levels in the diet. Moreover, Tu et al. (211) reported that GH mRNA expression in pituitary shows the opposite trend compared to hepatic IGF-I mRNA. Pérez-Sánchez et al. (199) found that gilthead seabream fed on low protein diets had significantly higher GH, but lower hepatic GH binding and lower IGF-I levels than fish fed on higher protein diets. This situation resembles the one discussed previously in which fish under fasting shows diminished IGFs production and elevated GH. Therefore, low and very high dietary protein levels may influence the GH-IGF axis through the same mechanism as fasting, possibly by direct control of pituitary hormone secretion by circulating nutrients.

#### Lipids

Dietary lipids have also been shown to modulate fish growth. In several fish species, an increase in the dietary lipid content or a decrease in the protein/lipid ratio was shown to have a negative effect on growth, as reported for the Senegalese sole (212), the turbot (213), and the flounder (214). Although a few studies show the effect of dietary lipids on fish growth performance, there is little knowledge on their effect on the regulation of the GH-IGF system. In Senegalese sole, it was demonstrated that an increase in dietary lipid content increases the hepatic IGF-I mRNA expression, and this was inversely correlated with the somatic growth (215). In pejerrey, an increase from 10 to 21% in the dietary lipid amount generates a decrease in the hepatopancreatic GHR-II mRNA expression, while no changes in somatic growth were found (216). In largemouth bass, the effects of different carbohydrates/lipids (CHO/L) ratios on the GH-IGF system, as well as on the somatic growth, were studied. Authors found that CHO/L ratios from 0.32 to 2.36 significantly upregulate GH mRNA expression and downregulate IGF-I mRNA expression. Higher ratios did not exert any further effects on them. A positive correlation between hepatic IGF-I mRNA levels and specific growth rates with varying dietary CHO/LIP ratios was found (217). All these findings indicate that dietary lipid level can differentially regulate the growth endocrine axis, at least at the transcription level. That can be directly associated with the role of these hormones in regulating lipid homeostasis, and particularly with the direct lipolytic effects of GH and the promotion of tissue growth by IGF-I. Moreover, results presented here also indicate that hepatic IGF-I mRNA as well as its plasma abundance could be a reliable index to assess growth and nutritional fitness.

Several studies have been focused on the effect of fatty acids present in dietary lipids and in the replacement of dietary fish oil by vegetable oil on fish growth. In most of the cases, an increase in the dietary amount of fatty acids from vegetable sources causes an increase in GH mRNA expression and/or circulating levels, while diminishes the IGF-I plasmatic and GHR-I mRNA levels (179, 203, 218). The hepatic mRNA expression of GHR-II and IGF-II seems to be constitutive and not affected by dietary fatty acids in sea bream (218), while in pejerrey the replacement of dietary fish oil by sunflower oil increases their mRNA expression (179). As a general conclusion, large replacements of high-unsaturated fatty acids by low-unsaturated fatty acids in fish diet lead to the GH-IGF system pattern to resemble one observed during fasting.

#### Micronutrients

The effect of micronutrients on the expression of GH-IGF system components was not extensively studied. To the best of our knowledge there is only one work reporting an effect of vitamins on *igf-I* expression (219). In this, the authors found that 4% of dietary vitamins generate an increase in the IGF-I mRNA expression in sea bass larvae after 38 days of the experiment, compared with diets containing lower or higher amounts.

## Future Perspectives

The role of nutrition as a potent modulator of the endocrine system governing feeding behavior and growth in fish is described in this review. Aquaculture research is a topic of increasing interest due to the demand for sustainable food production. That makes it essential to summarize the knowledge generated in the last past years in order to identify the weak points and to determine the direction in which new studies should be focused. As described here, food availability and feed composition are two of the most influent external signals modulating appetite-regulating and growth modulatory hormones. Future research should, therefore, be focused on tapping into this knowledge, expanding it in depth, and exploring its use to enhance feeding and growth in fish. Such approaches will eventually lead to increased yield in cultured fish.

It is also interesting to note the species-specific differences in the effect of feeding on the expression of appetite-regulating hormones and the GH-IGF system. Such differences could be related to the feeding behavior of specific fish species, as the carnivorous, herbivorous and omnivorous fish tend to fall into three different groups of responses. This fact also reflects the flexibility of metabolic systems, considering that the same components present in all fish species could generate distinct responses from the same food stimuli. Therefore, it is critical to bear in mind that results obtained from one species of fish might not apply to another.

As a final consideration, it would be interesting to broaden our knowledge of the crosstalk between hormones regulating food intake and those regulating growth. For instance, GH and IGF-I not only regulate somatic growth but can at the same time modulate lipid and carbohydrate metabolism, respectively (220). Others, including ghrelin, could stimulate food intake (221), and also GH release from pituitary (222, 223). Likewise, it has been shown that leptin directly regulates the expression of IGFs and GHRs in fish hepatopancreas (224). These are a few examples of the crosslinks mentioned above, that serve to highlight the close interaction that exists between hormones controlling food intake and growth to ensure the proper growth and development of fish. Thus, comprehensive approaches to determine both metabolic and growth regulatory hormone responses to nutritional challenges are more desirable from an aquaculture perspective. Future research must focus to identify gaps in knowledge, including the ones identified above. In addition, the use of alternate feed ingredients, use of endogenous feeding and growth regulatory factors as feed additives, and employing hormones using targeted molecular and cellular approaches should be explored to modulate growth rate and yield in cultured species.

## Author Contributions

JB prepared larger sections and complied the manuscript. AB, LS, JR, and CV wrote smaller sections. SU provided the idea, outline, provided feedback, and corrected the manuscript.

### Conflict of Interest Statement

The authors declare that the research was conducted in the absence of any commercial or financial relationships that could be construed as a potential conflict of interest.
